# Antinematode Activity of Abomasum Bacterial Culture Filtrates against *Haemonchus contortus* in Small Ruminants

**DOI:** 10.3390/ani11061843

**Published:** 2021-06-21

**Authors:** Asfa Nazish, Fozia, Baharullah Khattak, Taj Ali Khan, Ijaz Ahmad, Riaz Ullah, Ahmed Bari, Majid M. Asmari, Hafiz M. Mahmood, Muhammad Sohaib, Ahmad El Askary, Attalla F. El-kott, Mohamed M. Abdel-Daim

**Affiliations:** 1 Department of Microbiology, Kohat University of Science & Technology, Kohat 26000, Pakistan; asfanazish28@gmail.com (A.N.); baharkk75@gmail.com (B.K.); microbiologist63@yahoo.com (T.A.K.); 2 Boichemistry Department, KMU Institute of Medical Sciences, Kohat 26000, Pakistan; drfoziazeb@yahoo.com; 3 Institute of Pathology and Diagnostic Medicine, Khyber Medical University, Peshawar 25160, Pakistan; 4 Department of Chemistry, Kohat University of Science & Technology, Kohat 26000, Pakistan; abari@ksu.edu.sa; 5 Department of Pharmaceutical Chemistry, College of Pharmacy, P.O. Box 2457, King Saud University, Riyadh 11451, Saudi Arabia; masmary@windowslive.com; 6 Department of Pharmacology, College of Pharmacy, King Saud University, Riyadh 11451, Saudi Arabia; harshad@ksu.edu.sa; 7 Department of Soil Science, College of Food and Agriculture Sciences, King Saud University, P.O. Box 2460, Riyadh 11451, Saudi Arabia; msohaib@ksu.edu.sa; 8 Department of Clinical Laboratory Sciences, College of Applied Medical Sciences, Taif University, P.O. Box 11099, Taif 21944, Saudi Arabia; a.elaskary@tu.edu.sa; 9 Biology Department, Faculty of Science, King Khalid University, Abha 61421, Saudi Arabia; elkottaf@kku.edu.sa; 10 Zoology Department, College of Science, Damanhour University, Damanhour 22511, Egypt; 11 Pharmacology Department, Faculty of Veterinary Medicine, Suez Canal University, Ismailia 41522, Egypt; abdeldaim.m@vet.suez.edu.eg

**Keywords:** small ruminants, *H. contortus*, abomasum, fecal samples, bacterial culture filtrates

## Abstract

**Simple Summary:**

*Haemonchus contortus* is an important gastrointestinal nematode parasite of the tropical and sub-tropical regions that cause haemonchosis in small ruminants like goats and sheep. It causes low production, reduced growth and may cause death of the infected animals. Due to the resistance development and environmental issues, the use of anthelmintics can be replaced with biological control, which is an environment friendly alternative. In the present study, three bacteria viz; *Comamonas testosteroni*, *C. jiangduensis* and *Pseudomonas weihenstephanesis* showed significant effect on nematode mortality and egg hatch inhibition. It was also observed that the anthelmintic activity of these bacteria was dose dependent, where 100% bacterial metabolite concentration showed the highest activity. It is suggested that these bacteria may included in the integrated nematode management.

**Abstract:**

Haemonchosis is a parasitic disease of small ruminants that adversely affects livestock production. *Haemonchus contortus* is one of the most prevalent nematode parasites that infect the abomasum of small ruminants. This parasite reduces milk production, overall growth and sometimes causes the death of the infected animals. The evaluation of the biocontrol potential of some abomasum bacterial isolates against *H. contortus* is investigated in this study. Out of which, three isolates—*Comamonas testosteroni*, *Comamonas jiangduensis*, *Pseudomonas weihenstephanesis*—show significant effect against the nematode L3, adult, and egg hatch inhibition assays. Various concentrations of metabolites from these bacteria are prepared and applied in different treatments compared with control. In the case of adult mortality assay, 50% metabolites of *C. testosteroni* and *P. weihenstephanesis* show 46% adult mortality, whereas *C. jiangduensis* shows 40% mortality. It is observed that decreasing the concentration of bacterial metabolite, lowers nematode mortality. The minimum nematode mortality rate is recorded at the lowest filtrates concentration of all the bacterial isolates. The same trend is observed in egg hatch inhibition assay, where the higher concentration of bacterial culture filtrates shows 100% inhibition of *H. contortus* egg. It is concluded that the effect of bacterial culture filtrates against *H. contortus* is dose-dependent for their activity against nematode L3, adult, and inhibition of egg hatchment.

## 1. Introduction

Gastrointestinal parasites are considered as the main cause of economic losses in the livestock sector. Among gastrointestinal parasitic infections, haemonchosis is important and dominant that greatly destroys livestock production, particularly the small ruminants [1]. This disease is caused by three species of the genus Haemonchus, namely, *H. similis*, *H. placei*, and *H. contortus*. Among these, *H. contortus* is one of the most prevalent nematode parasites that infect the abomasum of small ruminants worldwide [2]. It is commonly known as a red stomach worm, the wire worm, or the barber’s pole worm. It belongs to phylum Nematoda, family Trichostrongyloidae, class Secernentea, and the order Strongylida. The highly susceptible part of ruminant’s stomach to *H. contortus* is the abomasum, in which adult worms are present. This parasite causes low production, decreased growth, lower body weight, and sometimes, cause the death of the infected host. This parasite is most prevalent in Africa; however, many cases have been reported in North America as well [3]. 

*Haemonchus contortus* mostly affects young animals, having hypo immunological response, showing low resistance to the parasite [4]. Primary symptoms of haemonchosisinclude pallor, anemia, edema, ill thrift, lethargy, and depression, which may cause sudden death in acute infection. Another prominent symptom of haemonchosis is the accumulation of fluid in the submandibular tissue, a phenomenon commonly called “bottle jaw” [5]. When the L3 larvae resume development in spring, the threat of haemonchosis increases. The young and pregnant or lactating mothers are highly susceptible to *H. contortus*, because of their low immunogenic response against the parasite infection [6]. A heavy infection (20,000–30,000 worms) of Haemonchus species can kill sheep and goats very quickly [7]. Haemonchosis can be diagnosed based upon the characteristic clinical signs of anemia, low Packed Cell Volume (PCV), pale mucous membranes dehydration, weakness, retarded growth, and edema. [8]. 

*Haemonchus contortus* is distributed throughout the world, where warm and humid climate prevails; hence, haemonchosis is a major threat in tropical and subtropical regions. [9]. Haemonchus species are prevalent in Pakistan and are reported almost from every district of Pakistan by different researchers with varying percent prevalence [10,11,12]. In Khyber Pakhtunkhwa and central Punjab, 72% prevalence of *H. contortus* was reported, while other researchers recorded its prevalence with varying percentages in many districts of Pakistan [13,14,15]. 

It is not advisable to eliminate the parasites from livestock, but to keep the population under a threshold, in a sustainable state [16]. To control the nematode parasites, different management practices, such as the use of chemical anthelmintics, sanitation, vaccination, various plant extracts, and biological control, are in common practice [17]. The helminths infection in man and animals is mostly treated by chemotherapy. Gastrointestinal nematode infections can be managed by using chemical anthelmintics, which are used as prophylactic measures. Due to the over-use of chemical anthelmintics, they reduce their effectiveness and emerge resistance in nematodes [18]. The *H. contortus* infection can also be effectively treated with the wire particles of copper oxide (COWP) and copper sulfate (CuSO_4_). Using copper oxide wire particles 2.5 g to 5 g in sheep, *H. contortus* eggs number was significantly reduced. These anthelmintics have been found to reduce the parasite population in small ruminants with low resistance development. Presently, it is used along with chemical antihelmintics to combat resistance development in *H. contortus* [19].

Limited information available regarding the anthelminthic activity of bacteria antagonists against parasitic nematodes. Some species, including *Bacillus* sp., have been reported to have nematocidal activity. A soil bacterium, *Bacillus thuringienisis*, is widely used as a biological control agent against different pathogens because of its low mammalian toxicity and the species specificity by particular endotoxin groups [20]. *Bacillus* sp. Produce a variety of toxic proteins that are vigorous against different parasites [21]. A number of toxins produced during vegetative growth show lethal activity; however, this lethal activity mainly results from the production of delta endotoxin that is synthesized during sporulation [22,23]. The production of other factors that might contribute to the toxic effects observed, such as proteases, chitinase, exotoxins, and lipases [24]. 

Little is known about the microbial diversity in the abomasum of the sheep, an important site of nematode infection, and the correlation between the microbial diversity and GIN resistance. Thus, this work aimed to isolate bacteria from the abomasum of sheep and goats to test the anthelmintic activity of metabolites of these bacterial isolates against *H. contortus*.

## 2. Materials and Methods

The present research work was carried out in the Department of Microbiology, Kohat University of Science and Technology Kohat. All the processes were performed in the aseptic environment. 

### 2.1. Collection of Abomasal Content and Fecal Samples

**Figure 1:**
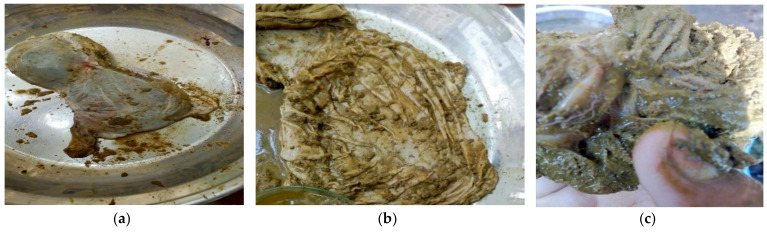
(**a**) Abomasum, (**b**) opened of abomasum, (**c**) adult *Haemonchus contortus* in abomasum.

Abomasum content, as shown in Figure 1a–c, was collected from slaughtered goats and sheep at a slaughterhouse in Kohat, Pakistan. A total of 50 samples of the abomasum and 50 samples of feces were taken from goats and sheep, and the samples were brought to Microbiology Laboratory for further processing. 

### 2.2. Isolation and Identification of Bacteria from Abomasal Contents

Bacteria were isolated by serial dilution method, taking abomasal fluid in 100 mL of distilled water. After that, 1 mL from the suspension was taken and put in a test tube containing 9 mL of distilled water. Different bacterial dilutions 10^−1^–10^−6^ and sterile distilled water (control) were used. Streaks were made from 10^−3^, 10^−4^, 10^−5^, and 10^−6^ bacterial dilutions on petri dishes containing nutrient agar. The plates were incubated at 37 °C for 24 h. The bacterial isolates were subcultured and identified by colony morphology and biochemical tests, and finally, through genomic DNA sequencing [25].

#### 2.2.1. Colony Morphology

Size, shape, colony consistency, margins, and elevation are included in the colony morphology.

#### 2.2.2. Gram Staining

Smear from each bacterial isolate was organized on a glass slide, and it was heat-fixed by passing it over the flame. Crystal violet was added drop by drop on the smear and left for 60 s. The slide was washed with tap water. For 45 s, iodine was overlaid on the slide and washed with distilled water. The slide was washed for 10 to 15 s with a decolorizer (alcohol). Smear was rinsed for few seconds with distilled water and counter-stained with safranin and air-dried. Deep violet or purple color emerged for Gram positive bacteria, while Gram negative bacteria appeared purple or red.

#### 2.2.3. Biochemical Characterization of the Bacterial Isolates

For the identification of bacterial isolates, various biochemical tests were performed, such as Oxidase test, Indole test, Sugar fermenter test, Motility test, Catalase test.

Catalase Test

A drop of hydrogen peroxide was added to a slide. A loop filled with each of the bacterial isolates was mixed into the droplet. Bubble formation indicated a positive catalase test. 

Oxidase Test

A smooth filter paper was put on a petri plate, and a drop of oxidase reagent was added. Bacterial culture was stretched on the droplet of the oxidase reagent, using an inoculating loop. The formation of the reagent’s dark purple color confirmed the oxidase positive test [26].

Indole Test

Bacterial colonies were inoculated into individual tubes of 2 mL tryptone water, incubated at 37 °C for 24 h, and tested for indole production with Kovac’s reagent. If the reagent showed a cherry red color layer, then it confirms the positive test.

Sugar fermenter

The sugar fermentation test was performed by inoculating a loop full of a nutrient broth culture of the organisms into the tubes containing different sugar media (five basic sugars, such as dextrose, sucrose, lactose, maltose, and mannitol) and incubated for 24 h at 37 °C. The sugar fermenter was shown by a color change from reddish to yellow and the formation of gas bubbles in the inverted test tubes.

Motility Test

One drop of bacterial culture, grown on nutrient broth, was placed on the coverslip. The same was placed inverted over around the concave depression of the hanging drop slide to make hanging drop preparation and sealed with Vaseline to prevent airflow and evaporation of the fluid. The hanging drop slide was then examined under 100× objective of a compound microscope using immersion oil. The motile and nonmotile organisms were identified by observing motility with to and from the movement of bacteria [27].

### 2.3. Isolation of Haemonchus Contortus Adults and Larvae from Abomasum

The abomasum of the freshly slaughtered goats and sheep were taken to pick adult *H. contortus* and larvae in the slaughterhouse of Kohat district, Pakistan. The worms were collected from the abomasum by washing with distilled water, and it was transferred into Phosphate Buffer Saline (PBS) with pH 7.4. The PBS was prepared by dissolving 8 g NaCl, 0.2 g KCl, 1.44 g Na_2_HPO_4_, and 0.24 g of KH_2_PO_4_ in 800 mL of distilled water, and distilled water was added to adjust the volume to one litter. 

### 2.4. Isolation of H. contortus Eggs from Faecal Sample

**Figure 2:**
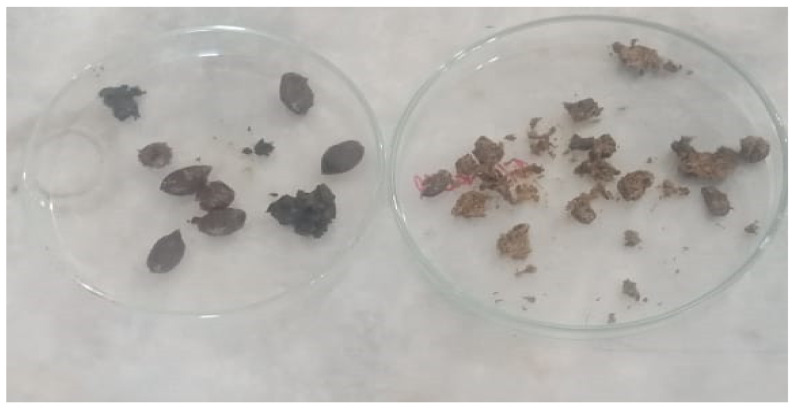
Fecal Samples of Sheep and Goats Containing *Haemonchus contortus* Eggs.

In a petri plate (Figure 2), about 4 g of feces were taken, and 60 mL floatation liquid was poured into the container by mixing feces carefully with a moving device. The resultant fecal suspension was poured over a filter of 200 µm into another vessel. The fecal contents were poured into a test tube. Then the test tube was topped gently with the interruption, a curved meniscus was formed at the top of the tube and placed a coverslip carefully. After 20 min, the droplet of liquid sticks to it. Under 10× and 40× of the compound microscope, the slides were observed [28].

#### 2.4.1. Estimation of Eggs in the Fecal Samples

The eggs of *H. contortus* in the animal’s fecal samples were calculated by using the McMaster counting chamber method [29]. In this procedure, Fresh feces of 3 g were taken and mix them in 42 mL of saturated sodium chloride solution. Through a tea filter or strainer, passed the suspension three times. Then both compartment of the McMaster counting chamber was filled with suspension and wait for 3–5 min and observed the McMaster chambers under the 10× of a light microscope.

Eggs seen in 1st and 2nd chambers were calculated as:

(Eggs seen in chamber 1 + Eggs seen in chamber 2) × 50 = Eggs per Gram (EPG)

The nematode eggs were taken from the feces of sheep and goats. Eggs from the highest EPG were isolated by dissolving 5 g of feces in 10 mL of distill water. The diluted feces were filtered through a 100 mesh and transferred into another flask. Then the saturated salt solution was added into filtrate in the threefold volume of the flask. On top of the container, placed a clear plastic sheet; hence, the surface of the solution could touch the plastic sheet and placed it for 60 min. The eggs were adhered to its lower surface, and it was carefully washed away by tap water in a clean container. By removing the upper layer of the water, the eggs were settled down at the end of the container after one hour [30].

#### 2.4.2. Culture Filtrate Collection from the Bacterial Isolates

The nutrient broth, containing 0.5% Peptone and 0.3% beef extract/yeast extract in distilled water, was autoclaved, and inoculated with bacterial isolates, and incubated in a shaking incubator at 37 °C for 7 days. The broth culture was centrifuged at 1000 rpm for 15 min. The pellet was discarded. With the help of Whatman filter paper, the supernatant was filtered and again filter through Miller HA syringe filters (pore size = 0.45 µm). The secondary metabolites were obtained. To check the presence of bacterial cells in metabolites, it was again inoculated on nutrient agar plates at 37 °C for 24 h. Extract of metabolites were mixed with phosphate buffer saline and distilled water to make different concentrations.

The extracts were considered as 100% concentrated, while the bacterial extracts with PBS and anthelmintic agent were considered as a positive control (PC), whereas only PBS and worms were considered as a negative control (NC).

### 2.5. In Vitro Bioassays

Various concentrations of the bacterial culture filtrates were used to evaluate their anthelmintic effect on adult and larval mortality and egg hatch inhibition of *H. contortus*, by using the standard techniques as per the protocol of Kotze [20].

#### 2.5.1. Adult Mortality Assay (AMA)

Adult worms were taken from the abomasum of freshly slaughtered sheep and goats to perform the adult mortality assay (AMA). Culture filtrate, obtained from three bacterial isolates Abomasum Bacteria with Pinkish Colony (ABP), Abomasum Bacteria with Yellow Colony (ABY), and Abomasum Bacteria with Creamy White Colony (ABCW), were diluted in PBS, as 100%, 50%, 25%, 12.5%, and 6.25% in 48 well plates, into which five adults’ nematodes were transferred into each well. The treatments were repeated five times. The bacterial culture filtrates were taken alone with worms, were considered as a 100% concentrated. For negative control, PBS with worms was used, whereas, for positive control, levasole (AgriLabs) 25 µg/mL in PBS with worms was used. Data on the adult nematode mortality were taken after every hour of the treatment, until all the worms were found dead in control. The treated worms were placed in warm PBS and observed their possible motility [31].

The percent nematode mortality was calculated by the following formula:

Percentage mortality = P test/P total × 100

P test: number of dead worms

P total: number of dead worms + number of live worms

#### 2.5.2. Larval Mortality Assay (LMA)

Larvae (L3) of *H. contortus* were taken from the abomasum of freshly slaughtered sheep and goats to perform the Larval Mortality Assay (LMA). Various concentrations of the bacterial metabolites (100%, 50%, 25%, 12.5%, and 6.25%) were prepared in PBS. The concentration was considered as 100% with L3 larvae and with metabolites. Well containing larvae and 2.5 mL phosphate buffer saline were considered as a negative control. The larvae to which an antihelmintic agent of 2.5 mL levasole (25 µg/mL) was added, considered as a positive control. The plates were placed in an incubator for 3 h at 37 °C. Under 10× of a stereo microscope, data on the larvae mortality were taken after three hours of treatments, until all the larvae were found dead in control. The treated worms were placed in warm PBS and observed their possible motility [32].

The percent larvae mortality was calculated as:

Percentage mortality of larvae = P check/P total

P check: number of dead larvae

P total: number of dead larvae + number of live larvae

#### 2.5.3. Egg Hatch Inhibition Assay

Egg hatch inhibition assay was performed to evaluate the inhibitory effect of the metabolites of the bacterial isolates ABP, ABY, and ABCW. This assay was repeated in triplicate following the protocol given by Coles et al. [33].

Nematode eggs were placed in 15 mL of sterile distilled water, and by the McMaster technique, their quantity was adjusted to 100–200 eggs in 75 µL of water and was added into each well of a 24 well titration plate. Metabolites of each bacterial isolates were added to each well at various concentrations of 100%, 50%, 25%, 12.5%, 6.25%, and 3.125%. Wells with nematode eggs, having no metabolites, were considered as a negative control, while 0.025 mg/mL Oxfendazole (Zenith Pharma, Karachi, Pakistan), in 0.3% DMSO, served as a positive control. The plates were incubated at 37 °C for 24 h. A droplet of Lugol’s iodine was added to maintain the process for 24 h. Under 10× of an inverted microscope, the total number of hatched and unhatched eggs were counted. The experiment was replicated five times [34].

The percent egg hatch inhibition was calculated as:

Inhibition of eggs = P test/P total × 100

P test: number of unhatched or hatched eggs.

P total: number of unhatched or hatched eggs + Larvae (L1)

### 2.6. Genomic DNA Extraction

DNA from the abomasal bacteria were extracted by the standard protocol of the phenol chloroform method. Fresh bacterial broth cultures were centrifuged at 13,000 rpm for 5 min. The supernatant was discarded, and pellets were resuspended in 550 µL of Tris-EDTA buffer with the addition of 30 µL of 10% SDS and 5 µL Proteinase K. Vortexed properly and incubated at 37 °C for 1 h. After incubation, 100 µL of 5 M NaCl and 80 µL of CTAB/NaCl were added and mixed properly and incubated again at 65 °C in a water bath for 10 min. An equal volume of phenol, chloroform, and isoamyl alcohol (25:24:1) was added, mixed properly, and centrifuged at 13,000 rpm for 5 min to purify DNA. The supernatant was transferred to a 1.5 mL fresh Eppendorf tube. To this tube, an equal volume of chloroform and isoamyl alcohol, was added and centrifuged at 13,000 rpm for 5 min. The supernatant was transferred to a 1.5 mL Eppendorf tube, and for the precipitation of DNA, 0.6 volume of chilled isopropanol was added and placed at −20 °C for 20 min. DNA was pelleted by centrifugation at 13,000 rpm for 5 min and washed the pellet with 70% ethanol, and then dried at room temperature. Finally, DNA was dissolved in 50 µL Tris-EDTA buffer overnight incubation at 37 °C. The DNA concentration was measured using a spectrophotometer by taking absorbance at 260 nm and diluted for polymerase chain reaction (PCR).

#### 2.6.1. DNA Confirmation and 16S RNA Amplification

The purified genomic DNA was verified through gel electrophoresis by mixing 4 µL genomic DNA with 2 µL loading dye and then load it in 1% agarose gel. The gel was run in a gel tank for 30 min at 120 volts and observed under UV transilluminator for DNA band. The amplification of the 16S RNA gene, universal primers, as shown in Table 1, were run on genomic DNA samples.

#### 2.6.2. PCR Conditions

The PCR tubes were put in the thermal cycle. The amplification was performed, following the condition given in Table 2.

#### 2.6.3. Gel Electrophoresis for PCR Products

The PCR products were confirmed on 1.5% agarose gel in TBE buffer containing 3 µL of ethidium bromide and run for 30 min at 100 voltages and 300 milli Ampere current. The bands were visualized by the Gel Doc system [35].

### 2.7. Statistical Analysis

The information acquired from the bioassays, i.e., adult nematode mortality, nematode larval mortality, and eggs hatch inhibitions assays, were evaluated by P Test via Statistic version 9.

## 3. Results

### 3.1. Isolation and Identification of Bacterial Isolates from Abomasal Contents

Three different colonies were subcultured based on different morphological characteristics.

#### 3.1.1. Colony Morphology

**Figure 3:**
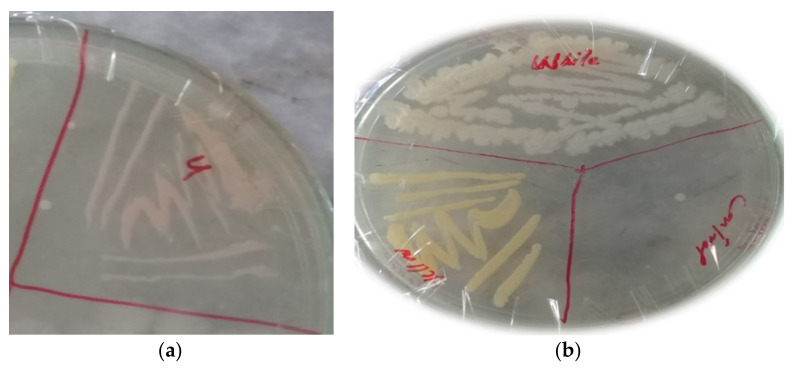
(**a**) Bacterial Isolate ABP colony; (**b**) bacterial Isolate ABY and ABCW colony.

The colony morphology of the bacterial isolates are shown in Table 3. The colonial morphology of the ABP bacterial isolate (Figure 3a appeared as irregular with an entire margin, and its elevation was flat and pinkish in color. Similarly, the colony morphology of ABY bacterial isolate was regular with a filamentous margin, and its elevation was flat and yellow. The colony morphology of the ABCW isolate was irregular in shape and creamy white, as shown in Figure 3b.

#### 3.1.2. Gram Staining

Gram staining revealed that all the bacterial isolates ABP, ABY, and ABCW, were Gram negative rods, as shown in Table 4.

#### 3.1.3. Biochemical Identification

Selected bacterial isolates ABP, ABY, and ABCW were identified by various biochemical tests as given in Table 4. Bacterial isolate ABP was negative for oxidase, catalase, and indole production, while positive for sugar fermenter and amotility test. The biochemical tests showed that the bacterial strain ABP was identified as *Comamonas testosteroni*. Bacterial isolate ABY was positive for catalase, oxidase test, and negative for motility test, indole, and sugar fermenter. Based on biochemical tests, the isolated bacterial strain ABY was reported as *Comamonas jiangduensis*. Bacterial isolate ABCW was positive for catalase and oxidase, while negative for sugar fermenter, motility test, and indole production. Based on the biochemical tests, the bacterial strain ABCW was considered as *Pseudomonas weihenstephanesis*.

### 3.2. In Vitro Bioassay

#### 3.2.1. Adult Mortality Assay (AMA)

**Figure 4:**
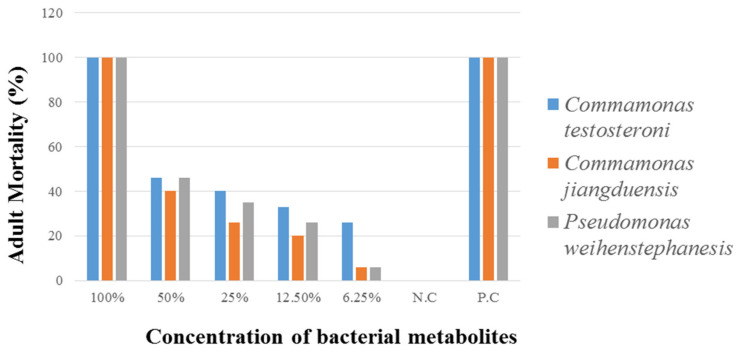
Percent adult nematode mortality by the bacterial isolates, after six hours of treatment.

Data regarding the mortality rate of *H. contortus* by various concentrations of bacterial culture filtrates are given in Table 5 and Figure 4. Bacterial isolate *C. testosteroni* caused the highest nematode mortality rate (100%) at 100% metabolite concentration. At 50% metabolite concentration, the nematode mortality was recorded as (46%). The lowest mortality rate (26%) was recorded at 6.25% metabolite concentration. Analysis of the data, regarding the adult nematode mortality by *C. jiangduensis*, showed that the bacterial metabolite had a significant effect on nematode mortality. The highest mortality rate (100%) was recorded at 100% concentration, followed by 50% metabolite concentration, where it was noted as 40%. While the minimum mortality rate (6%) was recorded at the metabolite concentration of 6.25%. As for the nematocidal effect of the bacterial isolate *P. weihenstephanesis* is concerned, the maximum adult nematode mortality rate (100%) was also recorded at 100% bacterial metabolite concentration. At 50% metabolite concentration, the mortality rate was recorded as 46%. The lowest adult nematode mortality of 6% was found at the lowest metabolite concentration, as with the case *C. jiangduensis*. The positive control in all cases showed the maximum activity of adult nematode mortality. While negative control shows no activity in all cases. Interestingly it was noted in all the above cases that the adult nematode mortality was found to be metabolite dosage-dependent.

#### 3.2.2. Larval Mortality Assay (LMA)

**Figure 5:**
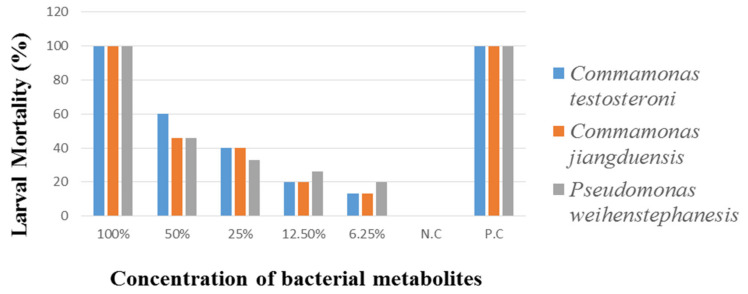
Percent nematode larval mortality by the bacterial isolates after six hours of treatment.

Larval Mortality Assay of *H. contortus*, was carried out on 3rd stage larva (L3) by treating with different concentrations of metabolites extract from *C. testosteroni*, *C. jiangduensis*, and *P. weihenstephanesis* with an exposure time of six hours. The results are given in Table 6 and Figure 5. Analysis of the data revealed that the highest 100% metabolite concentration of *C. testosteroni* caused 100% L3 mortality, followed by 60% with 50% metabolite concentration. The minimum nematode larval mortality (13%) was reported to be caused by the bacterial metabolite at a 6.25% concentration.

*Comamonas jiangduensis* showed 100% larval mortality at 100% bacterial metabolite concentration; at a 50% concentration the mortality was recorded as 46%, while the lowest dose (6.25%) of metabolite concentration caused the lowest (13%) mortality of L3 larvae. Analysis of the data showed that *P. weihenstephanesis* with 100% metabolite concentration caused 100% mortality of *H. contortus’* L3 larvae, which was followed by a 46% mortality rate, where 50% bacterial metabolite concentration was used. The lowest concentration of *P. weihenstephanesis* showed the lowest L3 mortality (20%), as in the case with the other bacterial isolates. The 100% concentration of all the bacterial isolates and the positive control showed the maximum L3 larva mortality, while the negative control showed no activity at all.

#### 3.2.3. Egg Hatch Inhibition Assay (EHA)

**Figure 6:**
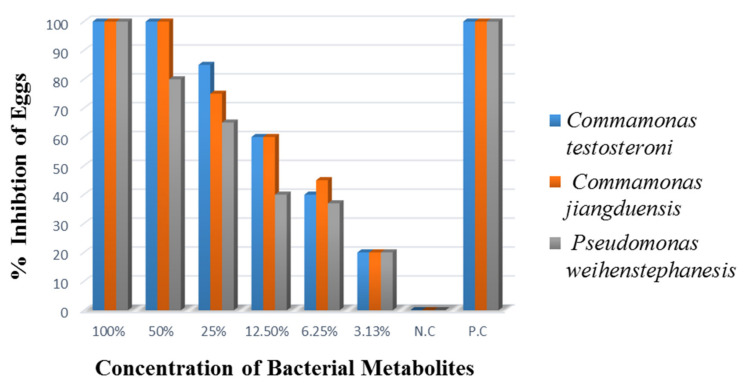
Percent Inhibition of eggs hatching, three days after treatment.

The results of egg hatch inhibition assay are presented in Figure 6. Analysis of the data showed that all the bacterial culture filtrates showed similar (100%) nematode egg hatch inhibition at 100% metabolite concentration. It was noted that the 50% metabolite concentration of *C. testosteroni* and *C. jiangduensis* inhibited 100% *H. contortus* eggs from hatching, while the same concentration of *P. weihenstephanesis* caused 80% nematode’s egg hatch inhibition. It was observed that lowering the bacterial metabolite concentration, lowers the egg hatch inhibition, and at the lowest metabolite concentration of 3.125%, showed the minimum (20%) egg hatch inhibition. The positive control (Oxfendazole) produced complete egg hatch inhibition, even at a very low concentration (0.025 mg/mL), while there was no egg hatch inhibition in the negative control.

### 3.3. Genomic DNA Extraction

**Figure 7:**
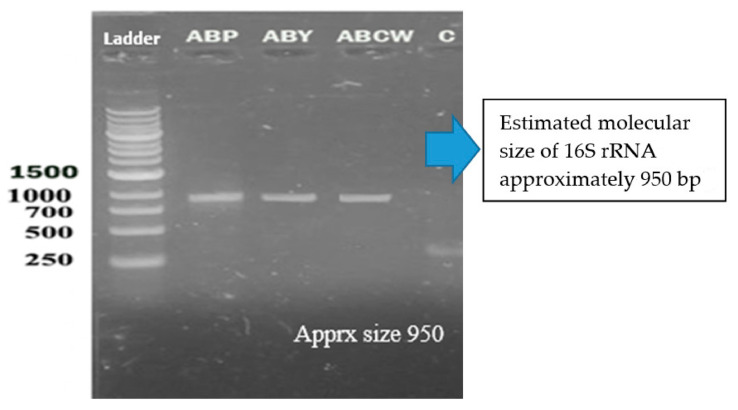
DNA bands of the bacterial isolates.

16S rRNA genes verified the bacterial isolates, and each gene fragment was effectively amplified and sequenced by a polymerase chain reaction from their DNA. On the gel, isolated groups of molecular DNA were noted for separate bacterial isolates. The recognized protein marker below 1 kb size was compared for molecular weight. The pattern of the band, found on agarose gel, revealed that the bacterial isolates ABP, ABY, and ABCW were nearly comparable and about 1000 bp of molecular weight, as shown in the Figure 7. The nucleotide sequences of three isolates were compared with the sequences of nearly linked isolates of 16S rRNA genes. The result revealed that ABP bacterial isolate showed resemblance to *Comamonas testosteroni* was provided by the server and ABY bacterial isolate showed resemblance to *Comamonas jiangduensis*, while ABCW bacterial isolate showed resemblance to *Pseudomonas weihenstephanesis* as provided by the server (Figure 7 and Table 7).

#### Phylogenetic Analysis

**Figure 8:**
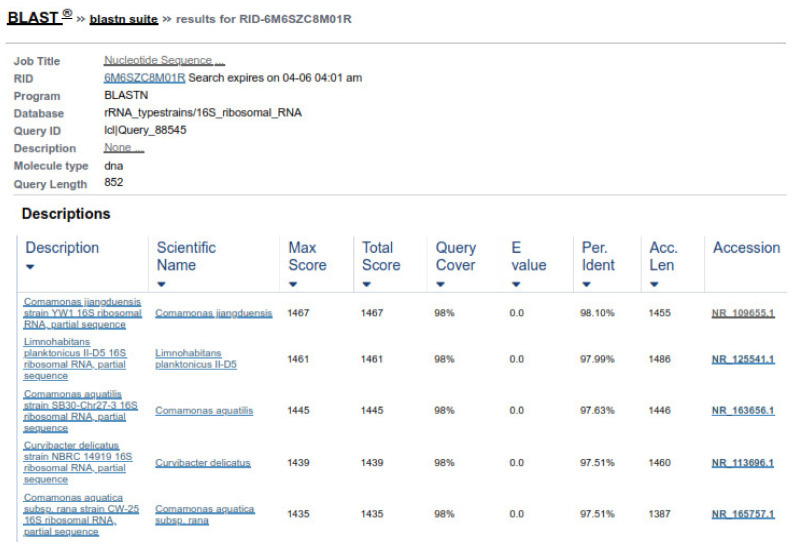
Sequence similarity of ABP with *Comamonas testosteroni*.

**Figure 9:**
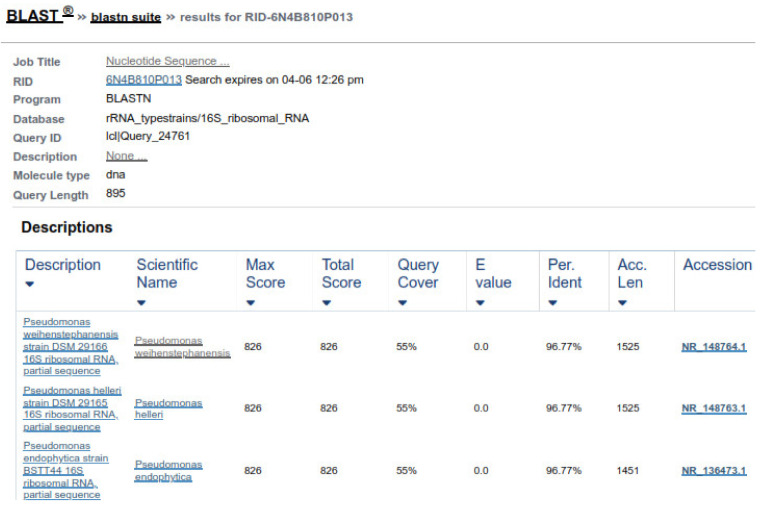
Sequence similarity of ABY with *Comamonas jiangduensis*.

**Figure 10:**
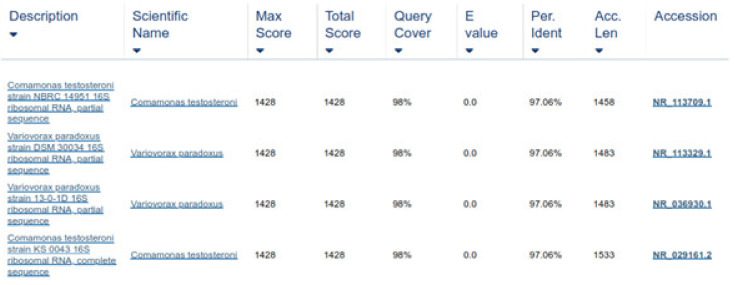
Sequence similarity less than 96% of ABCW, and hence, excluded.

Two forward and two reverse sequences for each sample were aligned using Bionumerics v3.5 (Applied Maths) to obtain a composite sequence. The quality of each sequence trace was visually assessed, and the poor-quality sequence was edited and removed. Organisms were identified for each assay, by comparing consensus sequences to a database library of known 16S rRNA gene sequences in GenBank (http://www.ncbi.nlm.nih.gov/blast/Blast.cgi, accessed on 3 April 2021) by multiple sequence alignment. The bacterial source of the sequence was identified by matching it with a sequence with the highest maximum identity score from the GenBank database. Where more than one bacterial species had the same highest score, all species were recorded in the results (Figure 8 and Figure 9). Sequences with 96% similarity to hits from the GenBank database were of poor quality and were excluded from this study (Figure 10).

**Figure 11:**
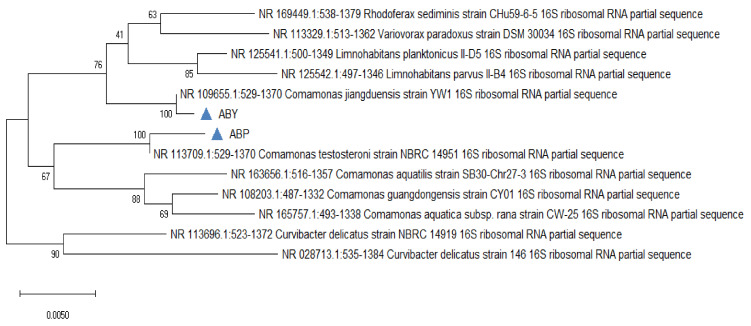
Phylogenetic Tree of *Comamonas testosteroni* (ABP) and *Comamonas jiangduensis* (ABY).

The phylogenetic tree was constructed on the origin of 16S rRNA gene sequences for the bacterial isolates using MEGA 6 software. Phylogenetic analysis showed (Figure 11) that ABP was identified as *Comamonas testosteroni* and ABY *Comamonas jiangduensis*.

## 4. Discussion

*Haemonchus contortus* has a great financial significance causing serious disease and death of cattle and ruminants [2]. Resistance to the available antihelmintic drugs has become a severe threat to livestock production [7]. To decrease the use of chemical anthelmintics, an alternative method is a biocontrol by using bacteria and fungi against the nematode parasites. *Duddingtonia flagrans* is as wide as nematophagous fungi, which is being explored to regulate intestinal nematodes in livestock [32]. *Bacillus thuringiensis* is one of the most commonly used bacterial antagonists in biological control of *H. contortus* that may promote insecticidal crystal proteins, commonly used to control pests and also due to its low mammalian toxicity [23].

The current study tried to explore the bacterial abilities to reduce the population of *H. contortus* at eggs, larval and adult stages. Bacterial isolates were collected from the abomasum of small ruminants. Different bacterial culture filtrate concentrations (100%, 50%, 25%, 12.5%, 6.25%, and 3.125%) were prepared and applied on three life stages of *H. contortus* to observe the mortality of adult, larvae, and egg hatch inhibition. Higher concentration 100% and 50% of *C. testosteroni*, *C. jiangduensis*, and *P. weihenstephanesis* showed 100% nematode eggs hatch inhibition. To our knowledge, these bacterial isolates have never been used against *H. contortus.* Some researchers worked on the effect of *Bacillus thuringiensis* on various life stages of *H. contortus* [30]. Earlier reports of leaf ethyl acetate and methanol extract of *A. squamosa*, *E. prostrata*, *S. torvum*, and *C. roseus* and acetone extract of *T. chebula* showed more consistent results on egg hatch inhibition of *H. contortus* [36].

Previous studies showed the larvicidal effect of various species of Bacillus, such as *Bacillus circulans* (Bcir), *B. thuringienisis* var. osvaldocruzi (Bto), *B. thuringienisis* var. israelensis (Bti), and *B. thuringienisis* var. kurstaki (Btk) on L3 stage of *Haemonchus* sp. among the tested bacteria, *B. circulans* and *B. thuringienisis* var. israelensis showed the best in vitro larvicidal efficiency of 90% and 94%, respectively, against the tested nematodes [37]. In our research studies, *H. contortus* larvae were treated with different bacterial metabolite concentrations, which cause 100% nematode mortality. These results are in line with the earlier reports on the control of nematodes in naturally infected sheep and goats, suggesting that the use of bacteria as an alternative control method for *H. contortus* larvae [21,37].

In the case of egg hatch inhibition assay, the highest bacterial culture filtrates of *C. testosteroni* and *C. jiangduensis* at 100% and 50% bacterial metabolite concentration, resulted in 100% inhibition of *H. contortus* eggs. The extract of the *Annona muricata* plant has also been used to inhibit the eggs hatch and mortality of *H. contortus* larvae and adults [38].

This study has extended the findings by showing that the tested bacteria species are effective against all stages of the nematode parasite. The metabolites of bacterial species *C. testosteroni* and *C. jiangduensis* showed a greater effect than filtrates, obtained from *P. weihenstephanesis*. Similarly, *C. jiangduensis* and *C. testosteroni* showed a higher mortality rate of L3 and adult nematodes as compared to *P. weihenstephanesis*. However, all the bacteria isolates showed a similar impact on eggs hatch inhibition. The positive control in all cases showed the maximum larva mortality, while the negative control exhibited no nematocidal activity at all. It was concluded that the larval and adult nematode mortality, as well as the nematode egg hatch inhibition, have a positive correlation with the doses or concentration of the metabolites, extracted from the bacterial isolates.

## 5. Conclusion

The present study was conducted to know the antihelmintic capabilities of metabolites extracted from abomasum bacteria *Comamonas testosteroni*, *C. jiangduensis*, and *Pseudomonas weihenstephanesis* against *H. contortus* eggs, larvae, and adults. It was noted that increasing the concentration of bacterial culture filtrates, increased nematode mortality. The same trend was observed in egg hatch inhibition assay bacterial culture filtrates. The effect of bacterial culture filtrates against *H. contortus* was found as dose-dependent. However, further in vivo bacterial culture filtrates investigation is recommended to seethe anthelmintic activity against various developmental stages of *H. contortus*.

## Figures and Tables

**Table 1:** Primer name and Sequence.

Primer	Sequence	Type
Forward	5-GGAGGCAGCAGTAGGGAATA-3	16s RNA (Universal)
Reverse	5-TGACGGGCGGTGAGTACAAG-3	16s RNA (Universal)

**Table 2:** PCR condition for 16s RNA universal primers.

Initial Denaturation		Denaturation		Annealing		Extension		Final Extension	
Temperature	Time	Temperature	Time	Temperature	Time	Temperature	Time	Temperature	Time
94 °C	5 min	94 °C	1 min	50 °C	40 s	72 °C	90 s	72 °C	5 min

**Table 3:** Colony Morphology and Gram Staining of Bacterial Isolates.

Bacterial Isolates	Colony Morphology			Gram Staining
	Color	Shape	Elevation	
ABP	Pinkish	Irregular	Flat	Gram Negative Rods
ABY	Yellow	Regular	Flat	Gram Negative Rods
ABCW	Creamy Yellow	Irregular	Flat	Gram Negative Rods

**Table 4:** Biochemical Tests for Bacteria Identification.

Bacterial Isolates	Oxidase	Indole	Sugar Fermenter	Motility	Catalase
ABP	Negative	Negative	Positive	Motile	Negative
ABY	Positive	Negative	Negative	Non motile	Positive
ABCW	Positive	Negative	Negative	Non motile	Positive

**Table 5:** Adult nematodes mortality by metabolite concentrations of the bacterial isolates.

Bacterial Metabolites Conc. (%)	Adult Nematodes Mortality (*n* = 15)			Standard Errors			*p*-Value		
	ABP	ABY	ABCW	ABP	ABY	ABCW	ABP	ABCW	ABY
100	5.0	5.0	5.0	-	-	-	-	-	-
50	2.3	2.0	2.3	0.33	0.57	0.33	0.01	0.01	0.07
25	2.0	1.3	1.6	0.57	0.88	0.66	0.07	0.12	0.26
12.5	1.6	1.0	1.3	0.88	0.57	0.66	0.19	0.18	0.22
6.25	1.3	0.3	0.3	0.33	0.33	0.33	0.05	0.42	0.42
N.C	-	-	-	-	-	-	-	-	-
P.C	5.0	5.0	5.0	-	-	-	-	-	-

Note: *p*-value ˂ 0.05 was considered significantly significant. NC: Negative Control; PC: Positive Control.

**Table 6:** Nematodes larval mortality is caused by various metabolite concentrations of the bacterial isolates.

Con. of Bacterial Metabolites (%)	Mean Nematode Larval Mortality (*n* = 15)			Standard Errors			*p*-Value		
	ABP	ABY	ABCW	ABP	ABY	ABCW	ABP	ABY	ABCW
100	5.0	5.0	5.0	-	-	-	-	-	-
50	3.0	2.3	2.3	-	0.88	0.33	-	0.11	0.01
25	2.0	2.0	1.3	-	0.57	0.33	-	0.07	0.05
12.5	1.0	1.0	1.3	-	0.57	0.66	-	0.22	0.18
6.25	0.6	0.6	1.0	0.66	0.66	-	0.42	0.42	-
N.C	-	-	-	-	-	-	-	-	-
P.C	5.0	5.0	5.0	-	-	-	-	-	-

Note: *p*-value ˂ 0.05 was considered significantly significant.

**Table 7:** Molecular Identification of bacteria strain.

Strain ID	Number of Nucleotides of 16S rRNA Gene	Closely Related Validly Published Taxa	Similarity (%) of 16S rRNA Sequence with Closely Related Species
ABP	832	*Comamonas testosteroni*	98.2
ABY	798	*Comamonas jiangduensis*	97.06
ABCW	856	*Pseudomonas weihenstephanesis*	96.0

## Data Availability

All available data incorporated in the MS.
